# The plant cell-wall enzyme AtXTH3 catalyses covalent cross-linking between cellulose and cello-oligosaccharide

**DOI:** 10.1038/srep46099

**Published:** 2017-04-26

**Authors:** Naoki Shinohara, Naoki Sunagawa, Satoru Tamura, Ryusuke Yokoyama, Minoru Ueda, Kiyohiko Igarashi, Kazuhiko Nishitani

**Affiliations:** 1 Plant Cell Wall Biology Laboratory, Graduate School of Life Sciences, Tohoku University, Aoba-Ku, Sendai, 980-8578, Japan; 2 Department of Biomaterial Sciences, Graduate School of Agricultural and Life Sciences, The University of Tokyo, Bunkyo-Ku, Tokyo, 113-8657, Japan; 3 Laboratory of Organic Chemistry, Department of Chemistry, Graduate School of Science, Tohoku University, Aoba-Ku, Sendai 980-8578, Japan; 4 VTT Technical Research Centre of Finland, P.O. Box 1000, Tietotie 2, Espoo FI-02044, Finland

## Abstract

Cellulose is an economically important material, but routes of its industrial processing have not been fully explored. The plant cell wall – the major source of cellulose – harbours enzymes of the xyloglucan endotransglucosylase/hydrolase (XTH) family. This class of enzymes is unique in that it is capable of elongating polysaccharide chains without the requirement for activated nucleotide sugars (e.g., UDP-glucose) and in seamlessly splitting and reconnecting chains of xyloglucan, a naturally occurring soluble analogue of cellulose. Here, we show that a recombinant version of AtXTH3, a thus far uncharacterized member of the Arabidopsis XTH family, catalysed the transglycosylation between cellulose and cello-oligosaccharide, between cellulose and xyloglucan-oligosaccharide, and between xyloglucan and xyloglucan-oligosaccharide, with the highest reaction rate observed for the latter reaction. In addition, this enzyme formed cellulose-like insoluble material from a soluble cello-oligosaccharide in the absence of additional substrates. This newly found activity (designated “cellulose endotransglucosylase,” or CET) can potentially be involved in the formation of covalent linkages between cellulose microfibrils in the plant cell wall. It can also comprise a new route of industrial cellulose functionalization.

Cellulose is the most abundant renewable biopolymer and has long been utilized by the human in the form of wood, paper, dietary fibre, etc. Functionalization of cellulose has huge potential for applications, and has been undertaken to develop new materials such as lightweight and durable composites, drug delivery matrices, electro-conducting elastic films, etc.^1^. However, cellulose modification routes are limited by the complex nature of cellulose (e.g, insolubility to water, heterogeneous crystallinity, and shape variations), and have been intensively studied with the aim of industrial applications^2^.

Plants produce cellulose in the form of insoluble microfibrils (or nanofibres; for terminology, see the ref. 1) embedded in soluble polysaccharides^3^,^4^. Of those polysaccharides, xyloglucan shares the β-1,4-glucan structure with cellulose, but is soluble in water presumably because of its side chain modifications^5^. Because xyloglucan non-covalently binds to cellulose^6^ and is long enough to interlink cellulose microfibrils^7^, currently prevailing models of the plant cell wall propose that cellulose microfibrils and xyloglucan form a structural network that can determine the physicochemical properties of the cell wall^3^,^4^,^8^,^9^.

Plant-specific extracellular enzymes of the xyloglucan endotransglucosylase/hydrolase (XTH) family have a unique activity: they seamlessly split and reconnect xyloglucan chains without the requirement for active donors (e.g., UDP-glucose)^10^,^11^,^12^. As such, those enzymes are postulated to mediate post-synthetic remodeling of the cellulose-xyloglucan network during plant growth^3^,^12^.

All the XTH enzymes belong to a larger group of glycoside hydrolase family 16 (or GH16^13^), and are distantly related to microbial endoglucanases^14^. Land plant genomes generally encode ~30 members of the XTH family^15^. By contrast, no XTH family members are found in algae^16^. Typical XTH family members in eudicots possess xyloglucan endotransglucosylase (XET) and/or xyloglucan endohydrolase (XEH) activity^10^,^11^,^17^,^18^, whereas some XTH family members found in angiosperms (AtXTH13, AtXTH14, and AtXTH18 of Arabidopsis^19^,^20^; HvXTH5 of barley^21^) and Pteridophyta (horsetail hetero trans β-glucanase or HTG of *Equisetum*^22^) catalyse heterogeneous transglucosylation between β-1,3/1,4-mixed-linkage glucan (MLG) and xyloglucan and/or between cellulose and xyloglucan. These activities are dubbed MLG:xyloglucan endotransglucosylase (MXE) and cellulose:xyloglucan endotransglucosylase (CXE), respectively^22^. In addition, nasturtium seeds contain a distinctive XTH protein (named *Tm*XET(6.3)) that shows broad substrate specificity and mediates endotransglucosylation from hydroxyethylcellulose (a synthetic soluble analouge of cellulose) to cello-oligosaccharide^23^.

Given that all XTHs utilize xyloglucan as the donor and/or acceptor substrate, the highly conserved nature of the XTH family members in the land plant lineage appears to contrast with the marked diversity of xyloglucan content in the cell walls of land plants^24^. Accordingly, it is conceivable that some XTHs might act on cellulose. Exploration of this possibility is important both to better understand how plants control the physicochemical properties of their cell walls during growth and for more efficient industrial cellulose processing. However, this has not been satisfactorily attempted, perhaps because of the difficulty in detecting the transglycosylation of cellulose.

Here, we report the development of assays for the detection of transglycosylation of cellulosic substrates, and biochemical characterization of Arabidopsis XTH proteins heterologously expressed in *Pichia pastoris*. We also performed phylogenetic analysis and database searches of XTH proteins. This report provides evidence that AtXTH3, a thus far uncharacterized member of the Arabidopsis XTH family, mediates transglycosylation of cellulosic substrates.

## Results

To estimate evolutionary history of the XTH family in land plants, we used protein sequences of all (33) XTHs identified in the genome of *Arabidopsis thaliana*; the sequence of barleyHvXET5, horsetail EfHTG, and poplar PtEG16; and some of their BLAST best hits from the wild grass (*Brachypodium distachyon*) and moss (*Physcomitrella patens*) genomes to construct a maximum-likelihood phylogenetic tree (Supplementary Fig. S1). EG16-like proteins, which belong to an evolutionary intermediate group between bacterial lichenases and plant XTHs^14^, were chosen as an outgroup. The XTH family was composed of two distinct clades, which were both conserved in the land plant lineage, and has previously annotated as the re-combined group I/II and the group III^14^. Each clade contained a subclade that did not include bryophyte taxa. Within the group I/II, the bryophyte-free subclade included four Arabidopsis XTHs (AtXTH1, AtXTH2, AtXTH3, and AtXTH11), and appeared to be biochemically uncharacterized; the other subclade contained AtXTH13, AtXTH14, AtXTH18, HvXET5, and EfHTG – which catalyse heterogeneous transglycosylation^19^,^20^,^21^,^22^, along with some AtXTHs (AtXTH12, AtXTH13, AtXTH14, AtXTH15, AtXTH17, AtXTH18, AtXTH19, AtXTH21, AtXTH22, AtXTH24, and AtXTH26) with XET activity^19^,^20^,^25^,^26^,^27^,^28^. Within the group III, the bryophyte-free subclade contained a member (AtXTH31) with XEH activity^29^, and the other subclade contained a member (AtXTH27) with XET activity^26^. For further analysis we chose AtXTH3 as a representative of the thus far uncharacterized subclade and AtXTH22 as a representative of the well-characterized clade that included AtXTH13, AtXTH14, AtXTH18, HvXET5, and EfHTG.

*AtXTH3* is highly up-regulated in a stem-growth–impaired mutant *acl5*^30^, whereas *AtXTH22* (also known as *TCH4*) has been isolated as one of the touch-inducible genes^31^. To obtain more information about the biological functions of those XTH proteins, we mined the microarray data collated in the eFP browser^32^, and found that *AtXTH3* was strongly expressed in the androecium, while *AtXTH22* was strongly expressed in expanding leaves. We also analysed gene expression patterns of other closely-related *XTH* genes in the eFP browser, and found that gene expression patterns were quite diversified even within a subclade. For example, *AtXTH2* was highly expressed in developing embryos, and *AtXTH21* was preferentially expressed in the root. We then observed a T-DNA insertion disruptant line (SALK_032898) of *AtXTH3*, and found no visible phenotype.

To directly test the possibility that some members of Arabidopsis XTHs might act on cellulose, we prepared recombinant AtXTH3 and AtXTH22 that were expressed heterologously in *P. pastoris* and purified by nickel-affinity chromatography (Supplementary Fig. S2), and used in transglycosylation assays. Transglycosylation involves two classes of substrates, i.e., donors (cleaved) and acceptors (conjugated to the nascent reducing end). As the donor substrates, we used xyloglucan and cellulose (in amorphous and crystalline forms); as the acceptor substrates, we used fluorescently-labeled (aminopyridyl; AP) derivatives of cellotetraose, laminaritetraose, and two xyloglucan oligosaccharides, *XXXG* and *XLLG*, (for nomenclature, see ref. 33). Since cellulose is insoluble in aqueous solution, cellulose preparations after reaction with XTHs were digested with a highly purified fungal cellulase preparation after removal of unreacted acceptor oligosaccharides, and the transglycosylation activity was determined by means of liquid chromatography-based measurements (Supplementary Fig. S3). Xyloglucan after reaction with XTHs was directly subjected to size exclusion chromatography-based enzyme activity measurements, as previously described^34^.

**Figure 1:**
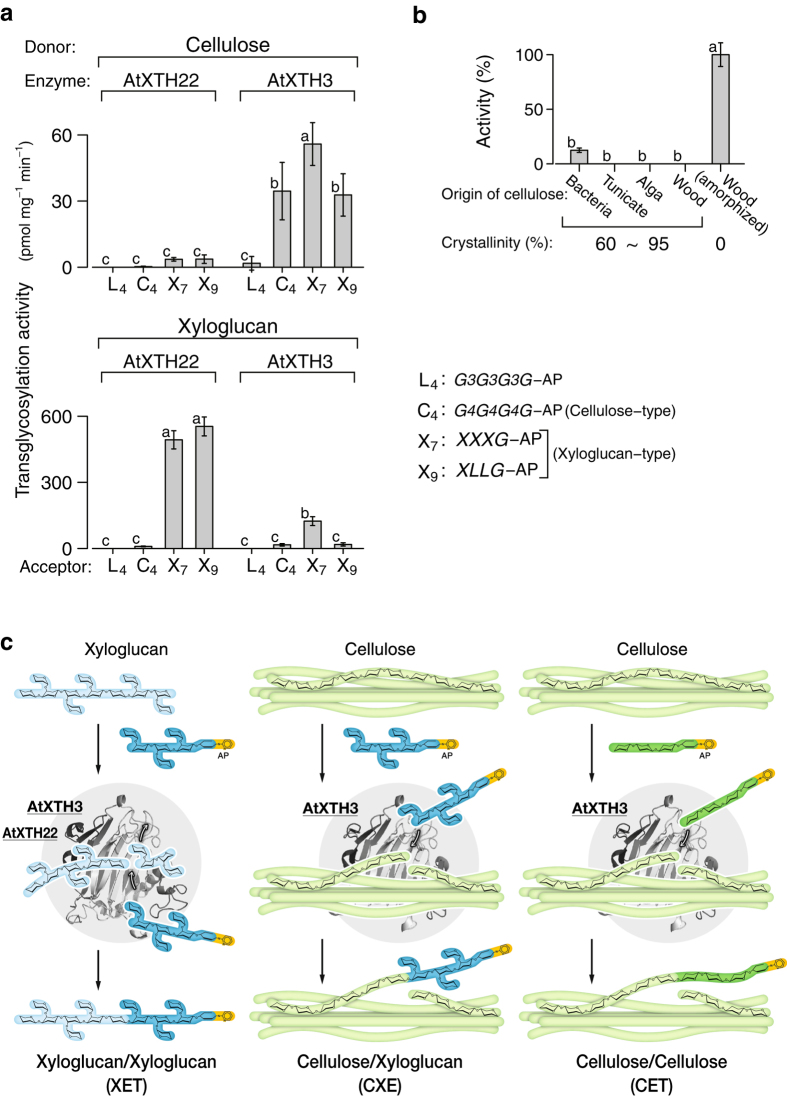
Substrate specificity of AtXTH3. (**a**) Transglycosylation activity from amorphous cellulose (top) or xyloglucan (bottom) to aminopyridyl (AP) oligosaccharides (L_4_: laminaritetraose; C_4_: cellotetraose; X_7_: xyloglucan-heptasaccharide, *XXXG*; X_9_: xyloglucan-nonasaccharide, *XLLG*). (**b**) Comparison of the donor substrate preference during AtXTH3-mediated endotransglucosylation of cellulose preparations of different crystallinity. X_7_ was used as the acceptor substrate. In (**a**) and (**b**), the data are presented as the mean ± s.d. from three independent experiments, and different letters denote significant differences as determined by Tukey’s test (*p* < 0.05). (**c**) Schematic representations of the types of transglycosylation activity of AtXTH3 and AtXTH22: (left) xyloglucan endotransglucosylase, XET, which mediates transglycosylation between xyloglucan and xyloglucan oligosaccharides; (middle) cellulose:xyloglucan endotransglucosylase, CXE, which mediates transglycosylation between cellulose and xyloglucan oligosaccharides; (right) cellulose endotransglucosylase, CET, which mediates transglycosylation between cellulose and cello-oligosaccharide.

Our data (Fig. 1a) indicate that AtXTH3 mediated endotransglucosylation from cellulose to cello-oligosaccharide. We designate this activity as cellulose endotransglucosylase (CET). AtXTH3 also mediated endotransglucosylation from cellulose to xyloglucan oligosaccharides (i.e., by CXE activity) as well as that from xyloglucan to xyloglucan oligosaccharides (by XET activity). Among these three activities, XET was the highest (Fig. 1a). On one hand, AtXTH22 almost exclusively transferred xyloglucans to xyloglucan oligosaccharides (Fig. 1a). The donor activity of crystalline cellulose preparations in AtXTH3-mediated cellulose transglycosylation was strikingly reduced, indicating that AtXTH3 preferentially recognizes amorphous regions of cellulose (Fig. 1b). Figure 1c schematically summarizes the transglycosylation reactions of XET, CXE, and CET.

**Figure 2:**
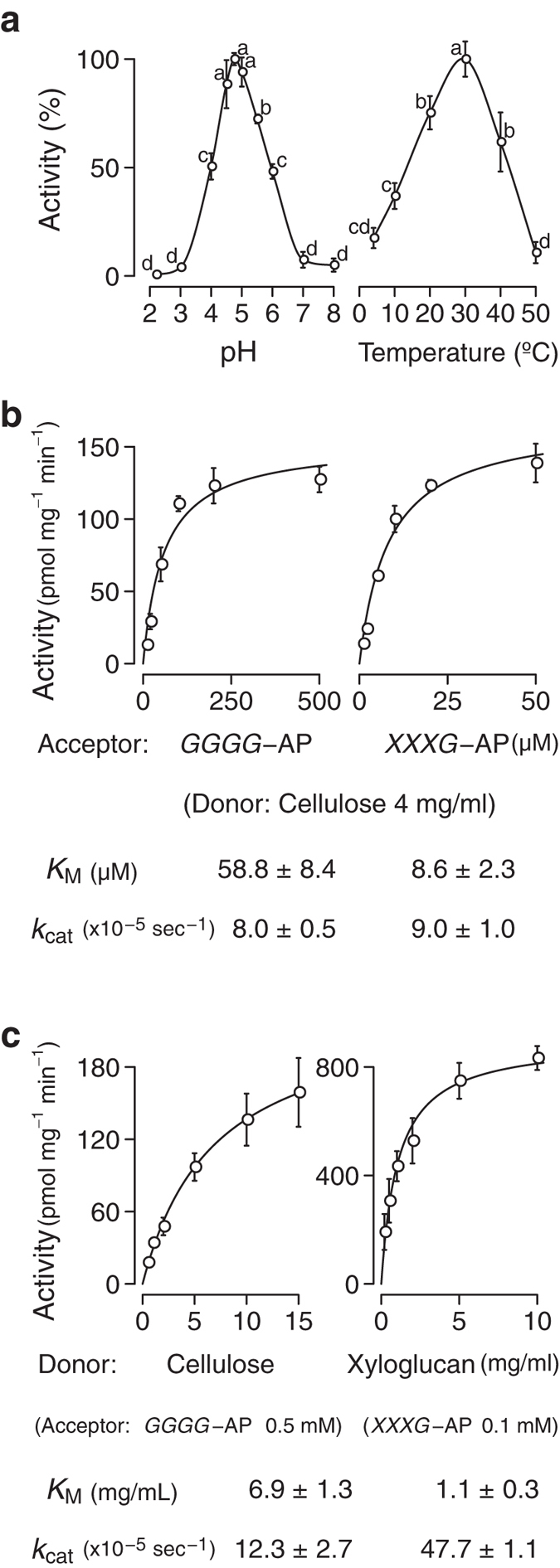
pH and temperature dependence and kinetic parameters of AtXTH3-mediated transglycosylation reaction. (**a**) pH (left) and temperature (right) dependence of AtXTH3-mediated transglycosylation (donor: amorphous cellulose; acceptor: aminopyridyl cellotetraose, *GGGG*-AP). (**b**) Michaelis constant (*K*_M_) and turnover number (*k*_cat_) for acceptor oligosaccharides (left: *GGGG*-AP; right: *XXXG*-AP) with amorphous cellulose as the donor substrate. (**c**) Michaelis constant and turnover number for donor substrates (left: cellulose donor and *GGGG*-AP acceptor; right: xyloglucan donor and *XXXG*-AP acceptor). The data are presented as the mean ± s.d. from three independent experiments. Different letters in (**a**) denote significant differences as determined by Tukey’s test (*p* < 0.05).

When pH and temperature dependence were examined, the CET activity of AtXTH3 was high at the pH range of 4.5–5.0 (Fig. 2a, left), and at 30 °C (Fig. 2a, right). The CET activity was most pronounced in the acidic pH range; this was in contrast with the pH optimum (5.5–6.5) for the XET activity of many AtXTH proteins examined^19^,^20^,^25^,^26^,^28^, but overlapped with the optimal pH (5.0) of the XET activity of AtXTH12^20^ and that (4.75) of the XEH activity of AtXTH31^35^, and fell within the apoplastic pH of plants^36^. When kinetic parameters of the CET were determined, the Michaelis constant was in the micromolar range for the acceptor (Fig. 2b) and in the milligram per milliliter range for the donor (Fig. 2c), and the turnover number was 8–12 × 10^−5^ sec^−1^ (Fig. 2b,c). Such turnover was more than 10 times slower than the XET and MXE activities of EfHTG^22^, but was on the same order as the XET activity of AtXTH3 (Fig. 2c).

**Figure 3:**
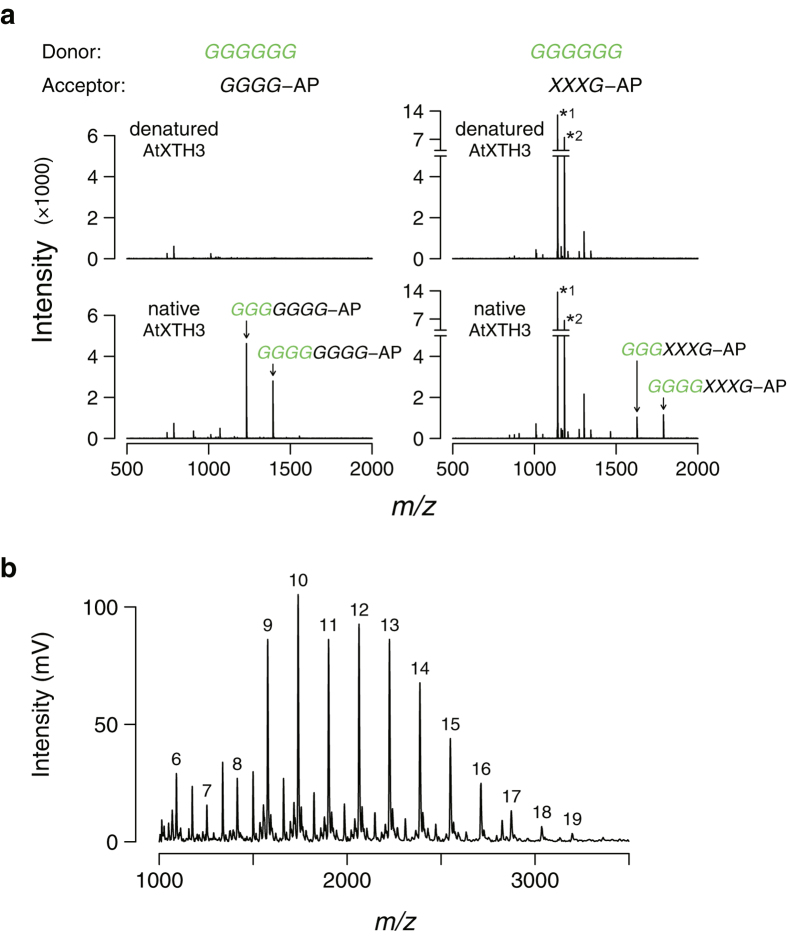
Characterization of AtXTH3 transglycosylation products. (**a**) LC/ESI-TOF mass spectra of reaction products generated in the course of AtXTH3-mediated transglycosylation (top: denatured; bottom: native) using cellohexaose (*GGGGGG*) as the donor substrate, and aminopyridyl cellotetraose (*GGGG*-AP) (left) or xyloglucan-heptasaccharide (*XXXG*-AP) (right) as the acceptor substrate. Two major peaks specifically detected in each native AtXTH3 preparation are annotated. The letters in green represent the structure of the donor substrate and its moiety transferred to the acceptor. The assay conditions for all reactions were set to remove the majority of the donor and acceptor substrates, although a fraction (less than 10%) of the acceptor remained (*1: [*XXXG*-AP + H]^+^; *2: [*XXXG*-AP + OAc + H]^+^). (**b**) MALDI-TOF mass spectrum of insoluble products of the AtXTH3-mediated transglycosylation where aminopyridyl cellohexaose (*GGGGGG*-AP) was the sole substrate. The numbers above peaks denote the degree of polymerization.

To investigate the mode of enzyme action, we used the soluble donor cellohexaose (instead of insoluble cellulose) and aminopyridyl cellotetraose and *XXXG* as the acceptors. The reaction products in reaction mixtures were detected directly by liquid chromatography–mass spectrometry. The mass spectra revealed that AtXTH3 mediated the transfer of three or four glucosyl residues from cellohexaose to the aminopyridyl acceptors (Fig. 3a).

Next, we examined whether AtXTH3 mediates the formation of cello-oligomers with a higher degree of polymerization (DP) or insoluble cellulose from soluble cello-oligosaccharides. To this end, aminopyridyl cellohexaose was incubated as a sole substrate with AtXTH3 for 168 h. AtXTH3 generated an insoluble and cellulase-digestible product that accounted for 8% of the input substrate. MALDI-TOF mass spectrometry analysis revealed that the insoluble product contained oligomers with DP of up to 19 (Fig. 3b). This indicates that the CET activity of AtXTH3 was responsible for the repeated transfer of the cellulosic moiety to and/or from the insoluble cellulose.

**Figure 4:**
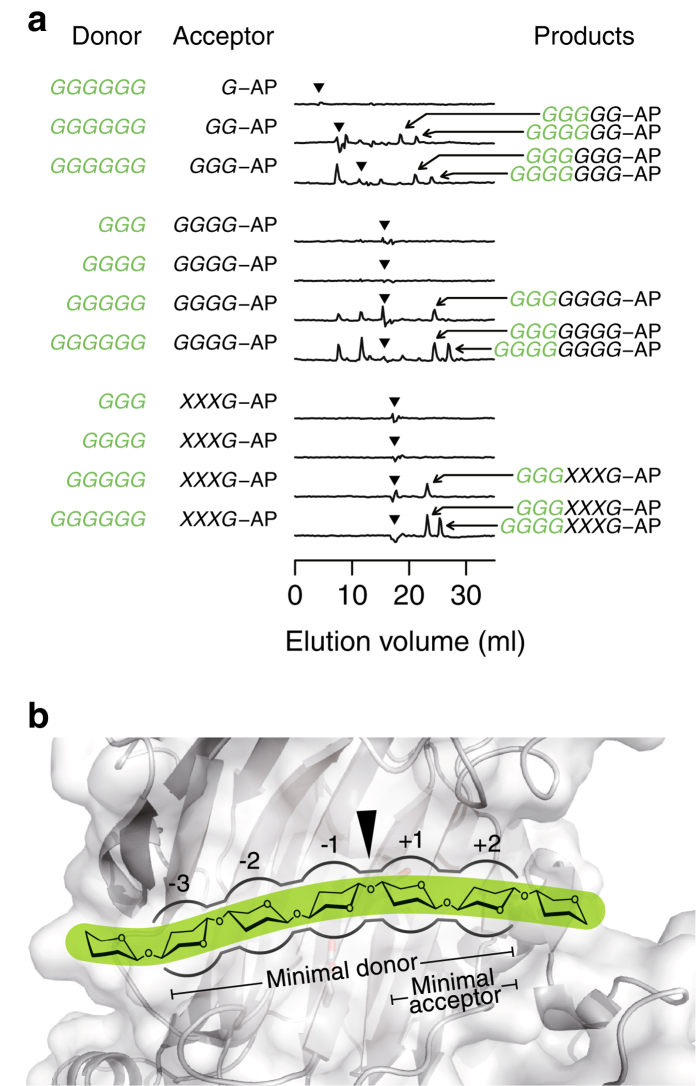
AtXTH3 subsite mapping. (**a**) Comparison of AtXTH3-mediated transglycosylation activities and reaction products when different combinations of donor/acceptor substrate combinations were used. Cellotriose (*GGG*), cellotetraose (*GGGG*), cellopentaose (*GGGGG*), and cellohexaose (*GGGGGG*) were used as the donors. Aminopyridyl glucose (*G*-AP), cellobiose (*GG*-AP), cellotriose (*GGG*-AP), cellotetraose (*GGGG*-AP), and xyloglucan-heptasaccharide (*XXXG*-AP) were used as the acceptors. The reaction products were separated by anion-exchange chromatography coupled with fluorescent detection. Each chromatogram is presented as the difference of chromatograms from reactions with native and heat-denatured enzymes. Arrowheads indicate acceptor elution volumes. Arrows indicate major transglycosylation products, whose putative structure is given on the right. The letters in green represent the structure of the donor substrate and its moiety transferred to the acceptor. (**b**) Schematic representation of the AtXTH3 subsite based on the data from (**a**). The 3D model was predicted using the PHYRE2 server^54^ with the amino acid sequence of AtXTH3 as a query; the template, found on the server, was the crystallographic data (1UMZ^56^) of PttXET16A, a hybrid aspen XTH protein.

To gain insight into the substrate-subsite recognition requirements, we evaluated the transglycosylation reaction using donor and acceptor oligomers with different DPs (Fig. 4a). Cellopentaose was the minimal donor of AtXTH3, from which cellotriose, but not cellotetraose, was transferred to the acceptor (Fig. 4a); aminopyridyl cellobiose was the minimal acceptor (Fig. 4a). These recognition patterns indicate that five contiguous β-1,4-glucosyl residues located at subsite positions −3 to +2 relative to the cleavage site are critical for the transglycosylation catalysed by AtXTH3 (Fig. 4b). These positions (−3 and +2) are also important for the XET reaction catalysed by a poplar XTH member PttXET16–34^37^, supporting the notion that CET and XET share a common reaction mechanism as well as a common protein framework.

## Discussion

Our results demonstrate that AtXTH3 cleaves the β-1,4-glucosidic linkage in amorphous cellulose and ligates the nascent reducing end to a non-reducing terminus of either cellulosic or xyloglucan oligosaccharide. We therefore discovered a plant enzyme that can mediate post-synthetic modification of cellulose. Plant cellulases, all of which belong to the GH9 family, are involved in such modification via partial hydrolysis of cellulose^38^. Nonetheless, unlike those cellulases, AtXTH3 can potentially mediate a grafting reaction from cellulose to cellulose.

The biological function of AtXTH3 is still far from clear. Because *AtXTH3* is up-regulated in *acl5*^30^, and was highly expressed in the androeciums, this protein might be involved in stem growth and androecium development. Nonetheless we did not detect any visible phenotypes of a knock-out mutant of *AtXTH3*. It has been pointed out that genetic and enzymatic redundancy potentially obstructs physiological analysis of individual XTH members^39^. We found that amorphous, but not crystalline, cellulose preparation was susceptible to AtXTH3. The crystallinity of cellulose has been estimated to be at ~20% in the primary cell wall^40^ and ~60% in the secondary wall^41^. In addition, the surface of cellulose microfibrils is likely to be composed of amorphous cellulose^42^,^43^. Considering that some members of Arabidopsis XTHs use synthetic analogues of cellulose (i.e., hydroxyethylcellulose and cellulose acetate) as donor substrates^20^, it is possible that the CET activity of AtXTH3, perhaps together with the same activity of some paralogue proteins, is involved in the formation of a covalent-linkage network on the surface of cellulose microfibrils, in concert with xyloglucan and the XET- and CXE- activities of XTH proteins.

For industrial applications, cellulose is processed and modified by mechanical, chemical, and enzymatic treatments^44^. The latter are advantageous because they can be performed under moderate conditions (i.e., ones that are cost and energy effective) and with high reaction specificities in reactions. However, enzymatic reactions applied to cellulose processing on a commercial basis are mainly confined to a cellulase-mediated partial digestion of cellulose^1^. This is presumably because of the paucity of known enzymes directly acting on cellulose with desirable specificity. On the other hand, effective modifications of cellulose are in high demand and many chemical methods have been developed^45^. In addition, surface modification of cellulose by a combination of chemically-modified xyloglucan-oligosaccharides and XET activity has been reported^46^. The discovery of the CET activity of AtXTH3 can comprise a new route for enzymatic cellulose modifications; potential advantages include the grafting specific for the β-1,4-glucosyl linkage and the simplicity of reaction with no requirement for additional substrates.

## Methods

### Preparation of enzymes

Recombinant AtXTH proteins were prepared using pPICZ vector (Invitrogen) modified so that the recombinant protein would have an N-terminal signal peptide of human serum albumin and a C-terminal 6 × His-tag. The partial amino acid sequences of AtXTH22 and AtXTH3, excluding their signal peptides, were subjected to codon-optimization for *P. pastoris*, and the optimized nucleotide sequences were then synthetized by a commercial DNA synthesis service (GenScript). The synthesized sequences were inserted into the modified vector. The expression vectors were introduced into *P. pastoris* KM71H cells (Invitrogen) by electroporation. Zeocin resistant colonies were screened as reported previously^47^. High expression clones were selected by using immunoblot analysis with an anti-His-tag antibody (H1029, Sigma-Aldrich). To evaluate the effect of *P. pastoris* intrinsic proteases on the accumulation of the proteins of interest (Supplementary Fig. S2), protein synthesis-inducing medium was prepared as described by the manufacturer (EasySelect *Pichia* Expression Kit; Invitrogen) and used without modification, or supplemented with protease inhibitor cocktail (0.04% v/v; P8215, Sigma-Aldrich). For large-scale production of recombinant proteins, AtXTH22 synthesis-inducing medium was used without modification, whereas the one for AtXTH3 production was additionally acidified (with K-phosphate buffer whose 1 M stock solution was adjusted to pH 3.0) and supplemented with K-EDTA (5 mM) and the protease inhibitor cocktail (0.04% v/v). The induction of recombinant protein expression was performed at 20 °C for 2–4 days with vigorous shaking and intermittent methanol feeding (0.5% v/v, every 24 h). The culture medium was then concentrated (~100 times) by ultrafiltration (10 kDa cutoff, Vivaspin Turbo 15, Sartorius). His-tagged proteins were purified from the concentrated medium using nickel-charged resin (cOmplete, Roche) and used directly in activity assays. Endoglycosidase H (New England Biolabs) was used as described in the manufacturer’s instructions.

*Trichoderma* cellulase was purchased from Megazyme and a recombinant PcCel6A cellulase was prepared as described previously^48^.

### Preparation of enzyme substrates

Tamarind xyloglucan (Megazyme) and microcrystalline cellulose preparations (Synaptech) were used as supplied; amorphous cellulose was prepared by treating cellulose powder (Wako Pure Chemical Industries) with phosphoric acid, as reported previously^49^. Cello-oligosaccharides, laminaritetraose, and xyloglucan oligosaccharides (Seikagaku) were used as supplied or coupled with 2-aminopyridine as described previously^50^. Since cellohexaose was largely insoluble in acetic acid, it was dissolved in hot (80 °C) dimethyl sulfoxide for coupling. Aminopyridyl oligosaccharides were purified using cellulose cartridges (Pyridylamination Manual Kit; Takara) or a gel-filtration column (Toyopearl HW-40F; Tosoh).

### Enzyme reactions

For polysaccharide-based transglycosylation assays, 40–100 μL of the reaction mixtures containing 2 mg/mL of amorphous cellulose or tamarind xyloglucan as donor substrates, 25 μM aminopyridyl oligosaccharide as the acceptor substrate, 1 μM (~30 μg/mL) recombinant AtXTH proteins, BSA (40 μg/mL), and 50 mM sodium acetate (buffered at pH 5.0 for AtXTH3 or pH 6.0 for AtXTH22) were incubated at 30 °C for 24 h, unless stated otherwise. For the control reaction, the enzymes were denatured by the addition of acetic acid (2% v/v) at the beginning of the incubation, or by heating at 90 °C for 5–15 min. When amorphous cellulose was used as the donor substrate, the reaction mixture was applied to a centrifugal filter unit (Ultrafree MC, 5 μm pore; Millipore) and washed four times with 50 mM sodium acetate buffer (pH 4.8); the retentate was suspended in the same volume of *Trichoderma* cellulase solution (12.5 U/mL) in 50 mM sodium acetate buffered at pH 4.8 and then incubated at 60 °C for 1 h, retaining the suspension on the filter; and the filtrate was collected by centrifugation and diluted up to 20 times in water or acetic acid (2% v/v) before liquid chromatography. The method was also modified as follows. For pH profile determination, McIlvaine buffer was used, as previously reported^21^. For kinetic parameter determinations, cellulose and xyloglucan donors (in the presence of 1 mg/mL BSA) were incubated with AtXTH3 and an acceptor substrate for 6 h and 3 h, respectively, to maintain first-order reaction conditions. When crystalline cellulose preparations were used as the donor substrates, 150 μg/mL of AtXTH3 was used and the cellulosic products were digested with 5 μM PcCel6A enzyme solution, pH 5.0, for 24 h.

For oligosaccharide-based transglycosylation assays, one or two oligosaccharides (100 μM each) were incubated with AtXTH3 under the same conditions as in polysaccharide-based transglycosylation assays except for the reaction duration (72 h). For mass spectrometry, the reaction mixture was extracted twice with a water-saturated phenol-chloroform (1:1, v/v) mixture and further purified on a MonoSpin NH2 column (GL Sciences).

To evaluate the formation of insoluble reaction products, 2 mM aminopyridyl cellohexaose was incubated with 20 μM AtXTH proteins, BSA (1 mg/mL), and thimerosal (0.01% w/v) in 50 mM sodium acetate (pH 5.0) for 168 h. Reaction mixture aliquot (100 μL) was then applied to an Ultrafree centrifugal filter unit (5 μm). For liquid chromatography, the retentate was washed four times with 50 mM sodium acetate buffer (pH 4.8), suspended in 100 μL of *Trichoderma* cellulase solution (12.5 U/mL in 50 mM sodium acetate buffer, pH 4.8), and then incubated at 60 °C for 3 h. For MALDI-TOF mass spectrometry analysis, the retentate was washed five times with water, and then suspended in 5 μL of water.

### Liquid chromatography

A Dionex ICS-5000 system equipped with pulsed-amperometric and fluorescence detectors was operated in two modes. Mode A: size exclusion chromatography (G5000PWXL–G3000PWXL in series; Tosoh) with isocratic elution (10 mM NaOH in 100 mM sodium acetate at a flow rate of 0.3 mL/min) was used to analyse samples containing the polysaccharide xyloglucan. Mode B: anion-exchange chromatography (Analytical CarboPac PA1 column; Dionex) with gradient elution (30–200 mM sodium acetate containing 100 mM NaOH, 0–40 min at a flow rate of 1 mL/min) was used to analyse samples containing cellulase-digested cellulosic reaction products or oligosaccharides.

The transglycosylation activity was determined by measuring fluorescence peak areas. In Mode A, fluorescence peaks eluted within 20 mL (following which the unreacted oligosaccharides were eluted) were identified as the transglycosylation products. In Mode B, fluorescence peaks found specifically in native enzyme samples were identified as the transglycosylation products. The peak areas were converted to molar activity by linear regression from a standard curve of the aminopyridyl oligosaccharide used in the reaction. To obtain subtracted chromatograms, each pair of chromatograms was aligned by linear time warping, and normalized on the basis of the sum of peak areas^51^.

The insoluble product generated by AtXTH3 was quantified using peak areas of cellulase-liberated glucose and cellobiose, with aminopyridyl cellohexaose that was digested in parallel as a standard.

### Mass spectrometry

Liquid chromatography system (1290 Infinity; Agilent) equipped with a reverse-phase column (Eclipse plus C18; Agilent) coupled to a mass spectrometer (MicrOTOF-II; Bruker) was used as an LC/ESI-TOF mass platform. The liquid chromatographic separation was performed with gradient elution (0.15 mL/min) of aqueous methanol acidified with acetic acid (0.05% v/v). Samples containing aminopyridyl cellotetraose were analysed using a linear gradient (5–18%, v/v, methanol, 0–5 min; Gradient A), while those containing aminopyridyl *XXXG* were analysed using a multi-step gradient (10–10–85–88–100%, v/v, methanol, 0–1.5–1.8–4.5–5 min; Gradient B). Mass spectra were recorded in the positive ion mode. The maximum intensities in retention time ranges (3–5 min for Gradient A; 3.7–4.3 min for Gradient B) were plotted against *m/z* values.

A MALDI-TOF mass instrument (AXIMA-CFR-plus; Shimadzu) was operated in linear positive ion mode. α-Cyano-4-hydroxycinnamic acid was dissolved (10 mg/ml) in aqueous acetonitrile solution (50% v/v) containing trifluoroacetic acid (0.1% v/v). An aliquot (0.5 μL) of the matrix solution was mixed with a 0.5 μL droplet of the analyte suspension on a target plate and then left to dry at room temperature.

### Computer-assisted sequence analysis

Amino acid sequences were retrieved from the TAIR database^52^ and the Phytozome database^53^. The 3D models based on amino acid sequences were predicted using the PHYRE2 server^54^. The phylogenetic tree was constructed with the SeaView software^55^ with the option of MUSCLE alignment; the subgroup annotation was based on the literature^14^.

## Additional Information

**How to cite this article:** Shinohara, N. *et al*. The plant cell-wall enzyme AtXTH3 catalyses covalent cross-linking between cellulose and cello-oligosaccharide. *Sci. Rep.* **7**, 46099; doi: 10.1038/srep46099 (2017).

**Publisher's note:** Springer Nature remains neutral with regard to jurisdictional claims in published maps and institutional affiliations.

## Supplementary Material

Supplementary Information

## References

[b1] Siró I. & Plackett D. Microfibrillated cellulose and new nanocomposite materials: a review. Cellulose 17, 459–494, doi: 10.1007/s10570-010-9405-y (2010).

[b2] Roy D., Semsarilar M., Guthrie J. T. & Perrier S. Cellulose modification by polymer grafting: a review. Chem. Soc. Rev. 38, 2046–2064, doi: 10.1039/b808639g (2009). 19551181

[b3] Carpita N. C. & Gibeaut D. M. Structural models of primary cell walls in flowering plants: consistency of molecular structure with the physical properties of the walls during growth. Plant J. 3, 1–30, doi: 10.1111/j.1365-313X.1993.tb00007.x (1993). 8401598

[b4] Cosgrove D. J. Growth of the plant cell wall. Nat. Rev. Mol. Cell Biol. 6, 850–861, doi: 10.1038/nrm1746 (2005). 16261190

[b5] Levy S., Maclachlan G. & Staehelin L. A. Xyloglucan sidechains modulate binding to cellulose during *in vitro* binding assays as predicted by conformational dynamics simulations. Plant J. 11, 373–386, doi: 10.1046/j.1365-313X.1997.11030373.x (1997). 9107029

[b6] Hayashi T. Xyloglucans in the primary cell wall. Annu. Rev. Plant Physiol. Plant Mol. Biol. 40, 139–168, doi: 10.1146/annurev.pp.40.060189.001035 (1989).

[b7] McCann M. C., Wells B. & Roberts K. Direct visualization of cross-links in the primary plant cell wall. J. Cell Sci. 96, 323–334 (1990).

[b8] Keegstra K., Talmadge K. W., Bauer W. D. & Albersheim P. The structure of plant cell walls III. A model of the walls of suspension-cultured sycamore cells based on the interconnections of the macromolecular components. Plant Physiol. 51, 188–197 (1973). 16658282 10.1104/pp.51.1.188 PMC367377

[b9] Park Y. B. & Cosgrove D. J. Xyloglucan and its interactions with other components of the growing cell wall. Plant Cell Physiol. 56, 180–194, doi: 10.1093/pcp/pcu204 (2015). 25613914

[b10] Fry S. C. . Xyloglucan endotransglycosylase, a new wall-loosening enzyme activity from plants. Biochem. J. 282, 821–828 (1992). 1554366 10.1042/bj2820821 PMC1130861

[b11] Nishitani K. & Tominaga R. Endo-xyloglucan transferase, a novel class of glycosyltransferase that catalyzes transfer of a segment of xyloglucan molecule to another xyloglucan molecule. J. Biol. Chem. 267, 21058–21064 (1992). 1400418

[b12] Rose J. K., Braam J., Fry S. C. & Nishitani K. The XTH family of enzymes involved in xyloglucan endotransglucosylation and endohydrolysis: current perspectives and a new unifying nomenclature. Plant Cell Physiol. 43, 1421–1435, doi: 10.1093/pcp/pcf171 (2002). 12514239

[b13] Lombard V., Golaconda Ramulu H., Drula E., Coutinho P. M. & Henrissat B. The carbohydrate-active enzymes database (CAZy) in 2013. Nucleic Acids Res. 42, D490–495, doi: 10.1093/nar/gkt1178 (2014). 24270786 PMC3965031

[b14] Eklöf J. M., Shojania S., Okon M., McIntosh L. P. & Brumer H. Structure-function analysis of a broad specificity *Populus trichocarpa* endo-β-glucanase reveals an evolutionary link between bacterial licheninases and plant *XTH* gene products. J. Biol. Chem. 288, 15786–15799, doi: 10.1074/jbc.M113.462887 (2013). 23572521 PMC3668736

[b15] Yokoyama R. . Biological implications of the occurrence of 32 members of the XTH (xyloglucan endotransglucosylase/hydrolase) family of proteins in the bryophyte *Physcomitrella patens*. Plant J. 64, 645–656, doi: 10.1111/j.1365-313X.2010.04351.x (2010). 20822502

[b16] Popper Z. A. . Evolution and diversity of plant cell walls: from algae to flowering plants. Annu. Rev. Plant Biol. 62, 567–590, doi: 10.1146/annurev-arplant-042110-103809 (2011). 21351878

[b17] Eklöf J. M. & Brumer H. The *XTH* gene family: an update on enzyme structure, function, and phylogeny in xyloglucan remodeling. Plant Physiol. 153, 456–466, doi: 10.1104/pp.110.156844 (2010). 20421457 PMC2879796

[b18] Hara Y., Yokoyama R., Osakabe K., Toki S. & Nishitani K. Function of xyloglucan endotransglucosylase/hydrolases in rice. Ann. Bot. 114, 1309–1318, doi: 10.1093/aob/mct292 (2014). 24363334 PMC4195539

[b19] Maris A., Suslov D., Fry S. C., Verbelen J. P. & Vissenberg K. Enzymic characterization of two recombinant xyloglucan endotransglucosylase/hydrolase (XTH) proteins of *Arabidopsis* and their effect on root growth and cell wall extension. J. Exp. Bot. 60, 3959–3972, doi: 10.1093/jxb/erp229 (2009). 19635745

[b20] Maris A. . Differences in enzymic properties of five recombinant xyloglucan endotransglucosylase/hydrolase (XTH) proteins of *Arabidopsis thaliana*. J. Exp. Bot. 62, 261–271, doi: 10.1093/jxb/erq263 (2011). 20732879

[b21] Hrmova M., Farkas V., Lahnstein J. & Fincher G. B. A barley xyloglucan xyloglucosyl transferase covalently links xyloglucan, cellulosic substrates, and (1,3;1,4)-*β*-D-glucans. J. Biol. Chem. 282, 12951–12962, doi: 10.1074/jbc.M611487200 (2007). 17329246

[b22] Simmons T. J. . Hetero-trans-β-glucanase, an enzyme unique to *Equisetum* plants, functionalizes cellulose. Plant J. 83, 753–769, doi: 10.1111/tpj.12935 (2015). 26185964 PMC4950035

[b23] Stratilová E. . Xyloglucan endotransglycosylases (XETs) from germinating nasturtium (*Tropaeolum majus*) seeds: isolation and characterization of the major form. Plant Physiol. Biochem. 48, 207–215, doi: 10.1016/j.plaphy.2010.01.016 (2010). 20153658

[b24] Vogel J. Unique aspects of the grass cell wall. Curr. Opin. Plant Biol. 11, 301–307, doi: 10.1016/j.pbi.2008.03.002 (2008). 18434239

[b25] Purugganan M. M., Braam J. & Fry S. C. The Arabidopsis TCH4 xyloglucan endotransglycosylase. Substrate specificity, pH optimum, and cold tolerance. Plant Physiol. 115, 181–190 (1997). 9306698 10.1104/pp.115.1.181 PMC158473

[b26] Campbell P. & Braam J. *In vitro* activities of four xyloglucan endotransglycosylases from *Arabidopsis*. Plant J. 18, 371–382, doi: 10.1046/j.1365-313X.1999.00459.x (1999). 10406121

[b27] Liu Y. B., Lu S. M., Zhang J. F., Liu S. & Lu Y. T. A xyloglucan endotransglucosylase/hydrolase involves in growth of primary root and alters the deposition of cellulose in *Arabidopsis*. Planta 226, 1547–1560, doi: 10.1007/s00425-007-0591-2 (2007). 17674032

[b28] Shi Y. Z. . Distinct catalytic capacities of two aluminium-repressed *Arabidopsis thaliana* xyloglucan endotransglucosylase/hydrolases, XTH15 and XTH31, heterologously produced in *Pichia*. Phytochemistry 112, 160–169, doi: 10.1016/j.phytochem.2014.09.020 (2015). 25446234

[b29] Zhu X. F. . *XTH31*, encoding an *in vitro* XEH/XET-active enzyme, regulates aluminum sensitivity by modulating *in vivo* XET action, cell wall xyloglucan content, and aluminum binding capacity in *Arabidopsis*. Plant Cell 24, 4731–4747, doi: 10.1105/tpc.112.106039 (2012). 23204407 PMC3531863

[b30] Tong W. . Thermospermine modulates expression of auxin-related genes in *Arabidopsis*. Front. Plant Sci. 5, 94, doi: 10.3389/fpls.2014.00094 (2014). 24672532 PMC3953664

[b31] Xu W. . Arabidopsis *TCH4*, regulated by hormones and the environment, encodes a xyloglucan endotransglycosylase. Plant Cell 7, 1555–1567, doi: 10.1105/tpc.7.10.1555 (1995). 7580251 PMC161010

[b32] Winter D. . An “Electronic Fluorescent Pictograph” browser for exploring and analyzing large-scale biological data sets. PloS one 2, e718, doi: 10.1371/journal.pone.0000718 (2007). 17684564 PMC1934936

[b33] Fry S. C. . An unambiguous nomenclature for xyloglucan-derived oligosaccharides. Physiol. Plant. 89, 1–3, doi: 10.1111/j.1399-3054.1993.tb01778.x (1993).

[b34] Nishitani K. A Novel Method for Detection of Endo-Xyloglucan Transferase. Plant Cell Physiol. 33, 1159–1164 (1992).

[b35] Kaewthai N. . Group III-A *XTH* genes of Arabidopsis encode predominant xyloglucan endohydrolases that are dispensable for normal growth. Plant Physiol. 161, 440–454, doi: 10.1104/pp.112.207308 (2013). 23104861 PMC3532273

[b36] Grignon C. & Sentenac H. pH and ionic conditions in the apoplast. Annu. Rev. Plant Physiol. Plant Mol. Biol. 42, 103–128, doi: Doi 10.1146/Annurev.Arplant.42.1.103 (1991).

[b37] Saura-Valls M. . Active-site mapping of a *Populus* xyloglucan *endo*-transglycosylase with a library of xylogluco-oligosaccharides. J. Biol. Chem. 283, 21853–21863, doi: 10.1074/jbc.M803058200 (2008). 18511421

[b38] Hayashi T., Yoshida K., Park Y. W., Konishi T. & Baba K. Cellulose metabolism in plants. Int. Rev. Cytol. 247, 1–34, doi: 10.1016/S0074-7696(05)47001-1 (2005). 16344110

[b39] Sampathkumar A., Neumetzler L. & Persson S. In The Plant Plasma Membrane (eds Murphy Angus S., Schulz Burkhard & Peer Wendy) 57–85 (Springer Berlin Heidelberg, 2011).

[b40] Chambat G., Karmous M., Costes M., Picard M. & Joseleau J. P. Variation of xyloglucan substitution pattern affects the sorption on celluloses with different degrees of crystallinity. Cellulose 12, 117–125, doi: 10.1007/s10570-004-1040-z (2005).

[b41] Andersson S., Serimaa R., Paakkari T., Saranpää P. & Pesonen E. Crystallinity of wood and the size of cellulose crystallites in Norway spruce (*Picea abies*). J. Wood Sci. 49, 531–537, doi: 10.1007/s10086-003-0518-x (2003).

[b42] Fernandes A. N. . Nanostructure of cellulose microfibrils in spruce wood. Proc. Natl. Acad. Sci. USA 108, E1195–E1203, doi: 10.1073/pnas.1108942108 (2011). 22065760 PMC3223458

[b43] Thomas L. H. . Structure of cellulose microfibrils in primary cell walls from collenchyma. Plant Physiol. 161, 465–476, doi: 10.1104/pp.112.206359 (2013). 23175754 PMC3532275

[b44] Khalil H. P. S. A., Bhat A. H. & Yusra A. F. I. Green composites from sustainable cellulose nanofibrils: A review. Carbohydr. Polym. 87, 963–979, doi: 10.1016/j.carbpol.2011.08.078 (2012).

[b45] Eyley S. & Thielemans W. Surface modification of cellulose nanocrystals. Nanoscale 6, 7764–7779, doi: 10.1039/c4nr01756k (2014). 24937092

[b46] Brumer H., Zhou Q., Baumann M. J., Carlsson K. & Teeri T. T. Activation of crystalline cellulose surfaces through the chemoenzymatic modification of xyloglucan. J. Am. Chem. Soc. 126, 5715–5721, doi: 10.1021/ja0316770 (2004). 15125664

[b47] Sunga A. J., Tolstorukov I. & Cregg J. M. Posttransformational vector amplification in the yeast Pichia pastoris. FEMS Yeast Res. 8, 870–876, doi: 10.1111/j.1567-1364.2008.00410.x (2008). 18637138

[b48] Igarashi K., Ishida T., Hori C. & Samejima M. Characterization of an endoglucanase belonging to a new subfamily of glycoside hydrolase family 45 of the basidiomycete *Phanerochaete chrysosporium*. Appl. Environ. Microbiol. 74, 5628–5634, doi: 10.1128/AEM.00812-08 (2008). 18676702 PMC2547050

[b49] Zhang Y. H., Cui J., Lynd L. R. & Kuang L. R. A transition from cellulose swelling to cellulose dissolution by *o*-phosphoric acid: evidence from enzymatic hydrolysis and supramolecular structure. Biomacromolecules 7, 644–648, doi: 10.1021/bm050799c (2006). 16471942

[b50] Hase S. Analysis of sugar chains by pyridylamination. Methods Mol. Biol. 14, 69–80, doi: 10.1385/0-89603-226-4:69 (1993). 8348245

[b51] Pierce K. M., Kehimkar B., Marney L. C., Hoggard J. C. & Synovec R. E. Review of chemometric analysis techniques for comprehensive two dimensional separations data. J. Chromatogr. 1255, 3–11, doi: 10.1016/j.chroma.2012.05.050 (2012). 22727556

[b52] Berardini T. Z. . The Arabidopsis information resource: Making and mining the “gold standard” annotated reference plant genome. Genesis 53, 474–485, doi: 10.1002/dvg.22877 (2015). 26201819 PMC4545719

[b53] Goodstein D. M. . Phytozome: a comparative platform for green plant genomics. Nucleic Acids Res. 40, D1178–1186, doi: 10.1093/nar/gkr944 (2012). 22110026 PMC3245001

[b54] Kelley L. A., Mezulis S., Yates C. M., Wass M. N. & Sternberg M. J. The Phyre2 web portal for protein modeling, prediction and analysis. Nat. Protoc. 10, 845–858, doi: 10.1038/nprot.2015.053 (2015). 25950237 PMC5298202

[b55] Gouy M., Guindon S. & Gascuel O. SeaView version 4: A multiplatform graphical user interface for sequence alignment and phylogenetic tree building. Mol. Biol. Evol. 27, 221–224, doi: 10.1093/molbev/msp259 (2010). 19854763

[b56] Johansson P. . Crystal structures of a poplar xyloglucan endotransglycosylase reveal details of transglycosylation acceptor binding. Plant Cell 16, 874–886, doi: 10.1105/tpc.020065 (2004). 15020748 PMC412862

