# Supplementary material for: The plant cell-wall enzyme AtXTH3 catalyses covalent cross-linking between cellulose and cello-oligosaccharide
Source: Sci Rep. 2017 Apr 26;7:46099. doi: 10.1038/srep46099 (PMC5405413; doi:10.1038/srep46099)
Supplement: Supplementary Information [file srep46099-s1.pdf]

Supplementary Information for

"The plant cell-wall enzyme AtXTH3 catalyses covalent cross-linking between cellulose and cello-oligosaccharide"

by Naoki Shinohara, Naoki Sunagawa, Satoru Tamura, Ryusuke Yokoyama, Minoru Ueda, Kiyohiko Igarashi, and Kazuhiko Nishitani

Supplementary Figures:

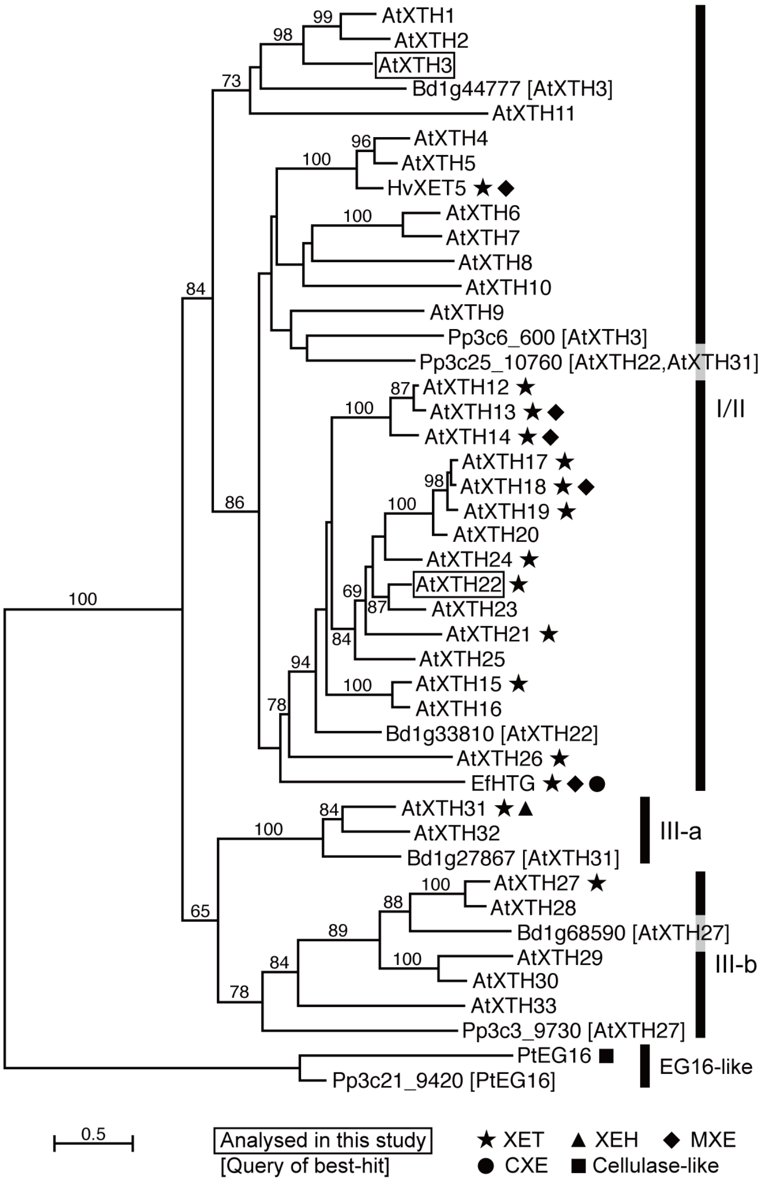

Supplementary Figure S1. Maximum-likelihood phylogenetic tree of XTH protein sequences. All XTH proteins (AtXTH1–AtXTH33) from *Arabidopsis thaliana*, barley (*Hordeum vulgare*) HvXET5, horsetail (*Equisetum fluviatile*)

EfHTG, and poplar (*Populus trichocarpa*) PtEG16, along with some of their BLAST best hits (queries are given in brackets) from the wild grass (*Brachypodium distachyon*) and moss (*Physcomitrella patens*) genomes, were included in the analysis. Previously reported enzymatic activities are annotated with symbols (star: xyloglucan endotransglucosylase; triangle: xyloglucan endohydrolase; diamond: β-1,3/1,4-mixed-linkage glucan:xyloglucan endotransglucosylase; circle: cellulose:xyloglucan endotransglucosylase; square: cellulase-like). Bootstrap values exceeding 65 out of 100 resampling replicates are shown.

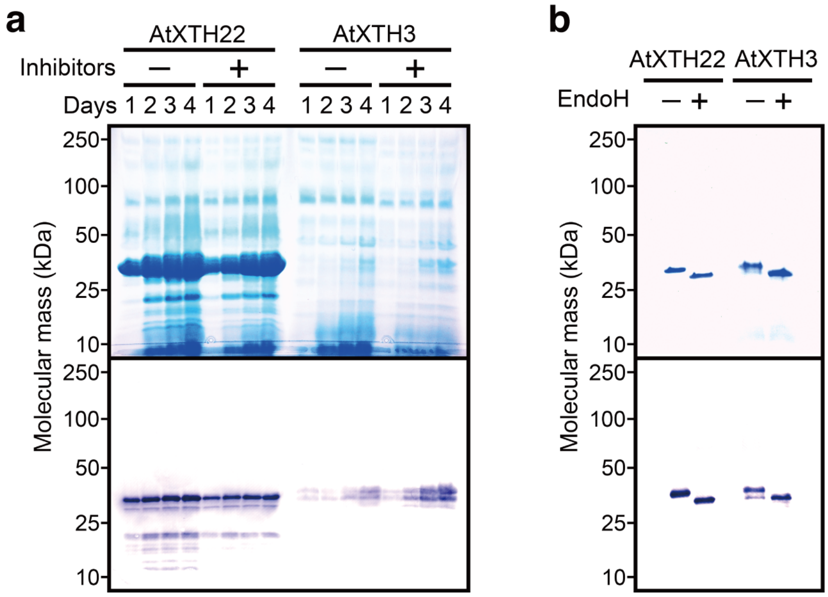

Supplementary Figure S2. Expression of the recombinant AtXTH proteins in *Pichia pastoris* and their purification on nickel-affinity columns. (a) Accumulation of AtXTH22 and AtXTH3 in *P. pastoris* culture medium was assessed by SDS-PAGE followed by Coomassie brilliant blue gel staining (top: each lane corresponds to protein from 1 mL of the culture medium) or by immunoblotting with anti-His-tag antibodies (bottom: AtXTH22 sample, protein from 10 μL of the culture medium; AtXTH3 sample, protein from 1 mL of the culture medium). The cells were cultured in the presence or absence of protease inhibitors. (b) The purity of nickel-affinity-purified AtXTH22 and AtXTH3 preparations was assessed by SDS-PAGE followed by Coomassie brilliant blue gel staining (top: 2 μg of protein per lane) or by immunoblotting with anti-His-tag

antibodies (bottom: 0.5 μg of protein per lane) with or without deglycosylation treatment with endoglycosidase H (Endo H).

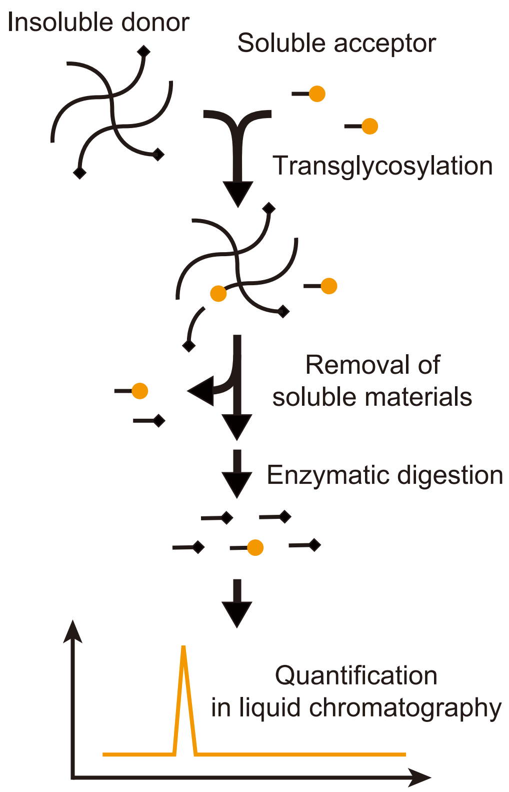

Supplementary Figure S3. Schematic representation of an insoluble polysaccharide-based transglycosylation assay. Solid lines: saccharide chains; closed squares: reducing ends; orange circles: fluorophore-modified reducing ends.
